# The effects of quercetin on seizure, inflammation parameters and oxidative stress in acute on chronic tramadol intoxication

**DOI:** 10.1186/s40360-021-00532-8

**Published:** 2021-10-19

**Authors:** Samaneh Nakhaee, Khadijeh Farrokhfall, Ebrahim Miri-Moghaddam, Mohsen Foadoddini, Masoumeh Askari, Omid Mehrpour

**Affiliations:** 1 Medical Toxicology and Drug Abuse Research Center (MTDRC), Birjand University of Medical Sciences (BUMS), Birjand, Iran; 2 Cardiovascular Diseases Research Center, Birjand University of Medical Sciences, Birjand, Iran; 3 Mel and Enid Zuckerman College of Public Health, University of Arizona, Tucson, AZ USA

**Keywords:** Acute on chronic, Inflammation, Oxidative stress, Quercetin, Tramadol

## Abstract

**Background:**

Tramadol is a widely used synthetic opioid for moderate to severe pain. Some studies have shown that tramadol can increase oxidative stress in different tissues of the body. Quercetin is also a substance with various biological effects, including antioxidant, anti-inflammatory, hepatoprotective, nephroprotective, and cardioprotective activities. The current investigation aimed at determining the effects of quercetin, with or without naloxone, on tramadol intoxication.

**Methods:**

This study was performed on 30 male Wistar rats divided into five groups: Group I) control group: intraperitoneal injections of normal saline 0.9% for 14 days; Group II) tramadol: 25 mg/kg for 14 days, and then a 50 mg/kg acute dose injection on the last day; Group III) acute quercetin (single dose): tramadol injection as with the second group plus 100 mg/kg of quercetin on the last day; Group IV) chronic quercetin: tramadol injection similar to the second group plus quercetin 100 mg/kg for 14 days; Group V) quercetin plus naloxone: tramadol injection similar to the second group plus injection of quercetin 100 mg/kg + intravenous naloxone 2 mg/kg on the last day, followed by a 4 mg/kg/h injection of naloxone for six hours. The rats were monitored for six hours on the last day, relating to the number and severity of seizures. Finally, the samples were prepared for biochemical investigation of the serum level of oxidative stress markers (MDA, SOD, NOx), inflammatory factors (IL-6, TNF-α), biochemical parameters (ALT, AST, creatinine, glucose) and hematological assay. The liver, heart, kidney, cortex, cerebellum, and adrenal tissues were collected to investigate the redox state.

**Results:**

None of the treatments had positive effects on the number and severity of seizures. Chronic administration of quercetin led to alteration of some blood parameters, including reduced hemoglobin level and elevated platelet counts. Acute on chronic tramadol administration resulted in a significant rise in AST, where different treatments failed to reduce their levels down to the control group.

**Conclusion:**

chronic administration of quercetin showed decreased oxidative/nitrosative stress in the liver, kidney, adrenal, and heart tissues. Quercetin plus naloxone decreased oxidative stress in the heart and adrenal tissues, but adverse effects on the brain cortex and hepatic function. Single-dose quercetin reduced cardiac oxidative stress.

## Background

Tramadol (TRM) is a central analgesic that is mostly used in moderate to severe pain. Its analgesic effect results from its having an agonist function on opioid receptors and inhibiting the reuptake of serotonin and norepinephrine (1, 2). Today, Tramadol has become one of the most widely used drugs globally, resulting in increased toxicity, complications, and mortality (3, 4). A tramadol overdose can lead to impaired consciousness, seizure and possible damages, agitation, respiratory depression, and serotonin syndrome (4, 5). Further, long-term use of this synthetic opioid is associated with addiction as well as physical and psychological dependence, which, despite therapeutic effects, can affect different organs of the body (3). Tramadol may cause self-limiting tonic-clonic seizures within 4–6 h after administration. Some pathways like inhibitory effects on gamma-aminobutyric acid (GABA) receptors, the serotonin, nitric oxide, opioid, and glutaminergic pathways, have been proposed in tramadol-induced seizures (4, 6).

Studies have shown that oxidative stress is one of the critical causes of damages in tramadol toxicity (6–8). Tramadol can induce a high regulation of lipid peroxidation, inflammation, and apoptosis markers. Also, it can alter neurotransmission in the rat cerebrum (9).

Furthermore, chronic consumption of it has shown histological disorders, such as increased apoptosis in the brain cortex of rats through developing oxidative stress (3, 10). In cases of tramadol poisoning, choosing the right therapy can be challenging. Naloxone, as an opioid antagonist, is used to prevent respiratory depression caused by non-synthetic opioids. However, there are contradictory studies about its usage in tramadol toxicity and its effect on seizures (11). In addition to typical chemical drugs that may be used for tramadol toxicity, other compounds that have shown beneficial effects in similar situations may prove helpful during toxicity with this widely used drug. Therefore, it will be substantially useful to design strategies to prevent toxicity with this drug on different systems and limit its medical complications. Quercetin is a flavonoid present in fruits and vegetables to which various therapeutic and protective properties have been attributed (12, 13). Quercetin has multiple advantageous effects against oxidative stress-related diseases. This substance has multiple biological effects due to its antioxidant, anti-inflammatory, hepatoprotective, and anti-apoptotic properties as well as neuroprotective potential (12, 13). The protective effects of this substance have also been shown on the heart (14–16).

Quercetin has several phenolic hydroxyl groups that have strong antioxidant activity. It has been reported to be a potent ROS cleanser that can modulate oxidative parameters (17). For instance, it enhances superoxide dismutase activities, catalase, glutathione peroxidase, and glutathione levels. Also, it can reduce the malondialdehyde’s serum levels. Besides, quercetin increased nitric oxide synthase activity. It also can increase nitric oxide levels in serum (18). Also, neuroprotective effects of quercetin on various central nervous system disorders like memory impairment (19, 20) and seizure (12, 13, 21) have been documented. The previous study results showed that quercetin reduced kainic acid-induced seizures by reducing GABA gene expression (12). Also, some earlier studies showed that quercetin has anti-seizure activity in acute and chronic models of kindling induced using pentylenetetrazole (PTZ) (13, 20). Chronic administration of quercetin (20–50 mg/kg/orally) in the case of ethanol withdrawal showed a protective effect against PTZ seizures (22). However, in another study, it was found that quercetin could not protect against seizures induced by N-methyl D-aspartate (NMDA) in rats. It has been suggested that quercetin may have proconvulsant effect (23). The protective effects of quercetin in lead, methylmercury, and tungsten toxicity have been reported (24). However, its protective effects concerning tramadol administration have remained understudied. Due to the positive effects of quercetin on seizures, its effects on the GABA receptor and its well-known systemic antioxidant/anti-inflammatory properties, the present study was designed to confirm the hypothesis that this phenolic compound can have a protective mechanism against tramadol-induced seizures. Also, the possible oxidant/antioxidant effect on other tissues and hematological parameters was monitored. Furthermore, the present study examined the effects of quercetin alone and naloxone (a commonly used opioid receptor antagonist in the case of tramadol-induced seizures) in acute tramadol administration.

## Methods

### Animals and materials

This study was performed on 30 male Wistar rats (body weight: 200–250 g, 12 weeks) procured from the department for keeping laboratory animals at Birjand University of medical sciences. The animals were subject to standard laboratory conditions (constant room temperature: 22 ± 2 °C), the light-dark cycle of 12 h, and free access to food and water. All experiments and treatments of rats were conducted according to the international laws of handling laboratory animals. The study protocol was also confirmed by the ethics committee of Birjand University of medical sciences (code: IR.BUMS.REC.1397.194). Sigma-Aldrich supplied quercetin (Q4951-10G), Naloxone (PHR1802-300MG), and pentobarbital (P3761-5G) while tramadol was purchased from Temad Co. (Iran, Karaj). Quercetin and naloxone were dissolved in DMSO while tramadol was in normal saline. The sample size calculation is the basis of the mathematical formula for “Group Comparison” design for animal studies (25). Healthy animals with normal behavior and activity from the same species, genders, weighing 200 to 250 g, and 12 weeks were included. Previously used rats in other experiments were not included. Exclusion criteria were considered as death during experiments and animals with aberrant behavior.

### Experimental design

After one week of adaptation to laboratory conditions, the animals were randomly assigned into five 6-member groups.

Group I (Control group): Normal saline 0.9%, intraperitoneally (IP) for 14 days.

Group II (Tramadol group): 25 mg/kg tramadol, IP for 14 days followed by acute administration of 50 mg/kg tramadol IP on the last day (the final dose of the last day: 75 mg/kg).

Group III (Acute quercetin group) (single dose): tramadol 25 mg/kg IP for 14 days. On the last day, acute IP injection of 50 mg/kg tramadol + quercetin 100 mg/kg IP following tramadol injection.

Group IV (Chronic quercetin group): 25 mg/kg tramadol IP + 100 mg/kg quercetin IP for 14 days, followed by 50 mg/kg tramadol IP on the last day (12, 13).

Group V (Quercetin plus naloxone group): 25 mg/kg tramadol IP for 14 days and on the last day, 50 mg/kg tramadol IP + 100 mg/kg quercetin IP + 2 mg/kg intravenous naloxone + 4 mg/kg/h naloxone for 6 h.

The rats were monitored for six hours on the last day for the number and severity of seizures. Seizure severity was rated according to the Racine criteria. In stage 1, rats were immobile, with closed eyes and facial clonus, whereas the hair around the nose was shaking. In stage 2, rats showed head-shaking accompanied by more severe facial clonus. In stage 3, rats experienced forelimb clonus. In stage 4, rats rose on their hind legs and exhibited bilateral forelimb clonus. In stage 5, rats rose on their hind legs, had no balance, fell, and underwent generalized tonic-clonic seizures (4). Video camera recorded all behavioral tests. A trained observer afterward analyzed videos. The researcher was aware of the rats’ experimental group, but a researcher, blinded to the group allocation, carried out statistical analysis and outcome assessment.

### Analyses of different parameters

Twenty-four hours after the last injection (for 12–14 h of fasting), the rats were sacrificed. 24 h after the end of the experiments, rats were profoundly anesthetized with 60 mg/kg sodium pentobarbital (Sigma-Aldrich, Germany, P 3761-5G) intraperitoneally before euthanization.

Then, blood specimens were taken from the heart. The serum was obtained through centrifugation and stored at − 20 °C until used. The EDTA-containing blood samples were used to determine complete blood count (CBC). The obtained serum was used to investigate biochemical (ALT, AST, creatinine, glucose), inflammatory (TNF-a, Interleukin-6), and oxidative stress (MDA, NOx, SOD) parameters. An IL-6 concentration was measured using a rat ELISA kit (ZellBio GmbH; Germany, Cat.No: ZB-10135C-R9648, the assay’s sensitivity: 2.5 ng/L). Also, Serum TNF-a was measured using a rat ELISA kit (Diaclone, France, Cat No:865.000.096); intra-and interassay coefficients of variations (CVs) were 8.2 and 6.5%, respectively. The liver, heart, kidney, cortex, cerebellum, and adrenal tissues were excised immediately and kept at − 80 °C until the time of analysis. The tissues were homogenized via a phosphate buffer cold solution (1:10, ph 7.7) by a homogenizer (MICCRA-D1, Germany) while being immersed in an ice bath. Next, the homogenated tissues were centrifuged at 15000 rpm for 20 min, where their supernatant was isolated. Homogenization and centrifugation were done carefully and in the same way in all samples. The serum and tissue concentration of NOx (nitrate and nitrite) was measured through the Griess reaction as previously described (26–28). As a byproduct of lipid peroxidation, the levels of thiobarbituric acid–reactive substances (TBARS) were determined using Uchiyama and Mihara’s method (29). MDA is the final product of the peroxidation of fatty acids, which creates a colored complex when combined with thiobarbituric acid (TBA). Based on the reaction with this acid at acidic pH at 90–100 °C, the absorption of the pink product resulting from the reaction was measured using spectrophotometry at the wavelength of 532 nm. A Nasdox™–Superoxide Dismutase Non-Enzymatic kit (Code: NS-15033, Navandsalamat, Iran) was employed to measure the level of superoxide dismutase (SOD). This test is based on inhibiting the pyrogallol autooxidation reaction, where the absorbance was read via an ELISA reader at the wavelength of 405 nm. It was then placed in the following formula as sample SOD activity (U/ml) $=\frac{\mathrm{OD}\ \mathrm{test}}{\mathrm{OD}\ \mathrm{control}}$ × 200. All samples of a test variable were assessed together to minimize the interassay coefficient of variation measurement.

### Statistical analysis

After collection, the data were introduced into SPSS 16. The assumption of data normality was investigated via the Shapiro Wilk test. In order to compare the means in the groups, the one-way ANOVA plus the Bonferroni post hoc test was used. In the case of abnormal data distribution, the Kruskal Wallis test and Dunn-Bonferroni test were employed. *P* < 0.05 was considered statistically significant. All data were included in the analysis.

## Results

### Biometrical data

In this study, one case of mortality was observed in the chronic quercetin (Group IV) and quercetin plus naloxone (Group V) groups. Before and after the intervention, the rats’ average weights were 218.08 ± 18.8 and 259.7 ± 34.6 g, respectively, whereas there was no difference between the groups before the study (*p* > 0.05). However, at the end of the study, the mean weight of the chronic quercetin group (Group IV) was significantly lower than that of the tramadol and control groups. Further, the chronic injection of quercetin for this group lowered the heart weight considerably compared to other groups (Table 1). Kidney and liver weight were similar in control, tramadol, and different treatment groups (Table 1). The ratio of tissue weight to body weight did not differ between groups. (*p* > 0.05).

**Table 1:** Weight of rats and organ weight in different experimental groups

Variables	Control	Tramadol	Tramadol + single Quercetin	Tramadol + chronic quercetin	Tramadol + single Quercetin + Naloxone
Weight of rats before intervention (g)	219.1 ± 9.02	216.8 ± 15.5	217.2 ± 21.5	216.7 ± 27.8	220.6 ± 18.2
Weight of rats after intervention (g)	271.7 ± 15.7	271.6 ± 20.8	265.2 ± 23.19	229.33 ± 33.7*,**	264.37 ± 4.52
Weight of liver (g)	9.1 ± 0.9	8.8 ± 1	8.05 ± 0.3	8.7 ± 0.3	9.4 ± 0.6
Weight of kidney (g)	1.7 ± 0.5	1.8 ± 0.1	1.8 ± 0.3	1.8 ± 0.1	1.8 ± 0.6
Weight of heart (g)	0.82 [0.79,0.88] †	0.85 [0.83,0.91] †	0.87 [0.83,0.88] †	0.75 [0.71,0.8]	0.86[0.86,0.86] †

Statistical comparison between groups was made using one-way ANOVA followed by Bonferroni posthoc or Kruskal Wallis test and Dunn-Bonferroni post hoc

*p* < 0.05 significant as compared to the **control group**

** *p* < 0.05 significant as compared to **tramadol group**

† *p* < 0.05 significant as compared to **Tramadol + chronic Quercetin group**

### Behavioral parameters

**Fig. 1:**
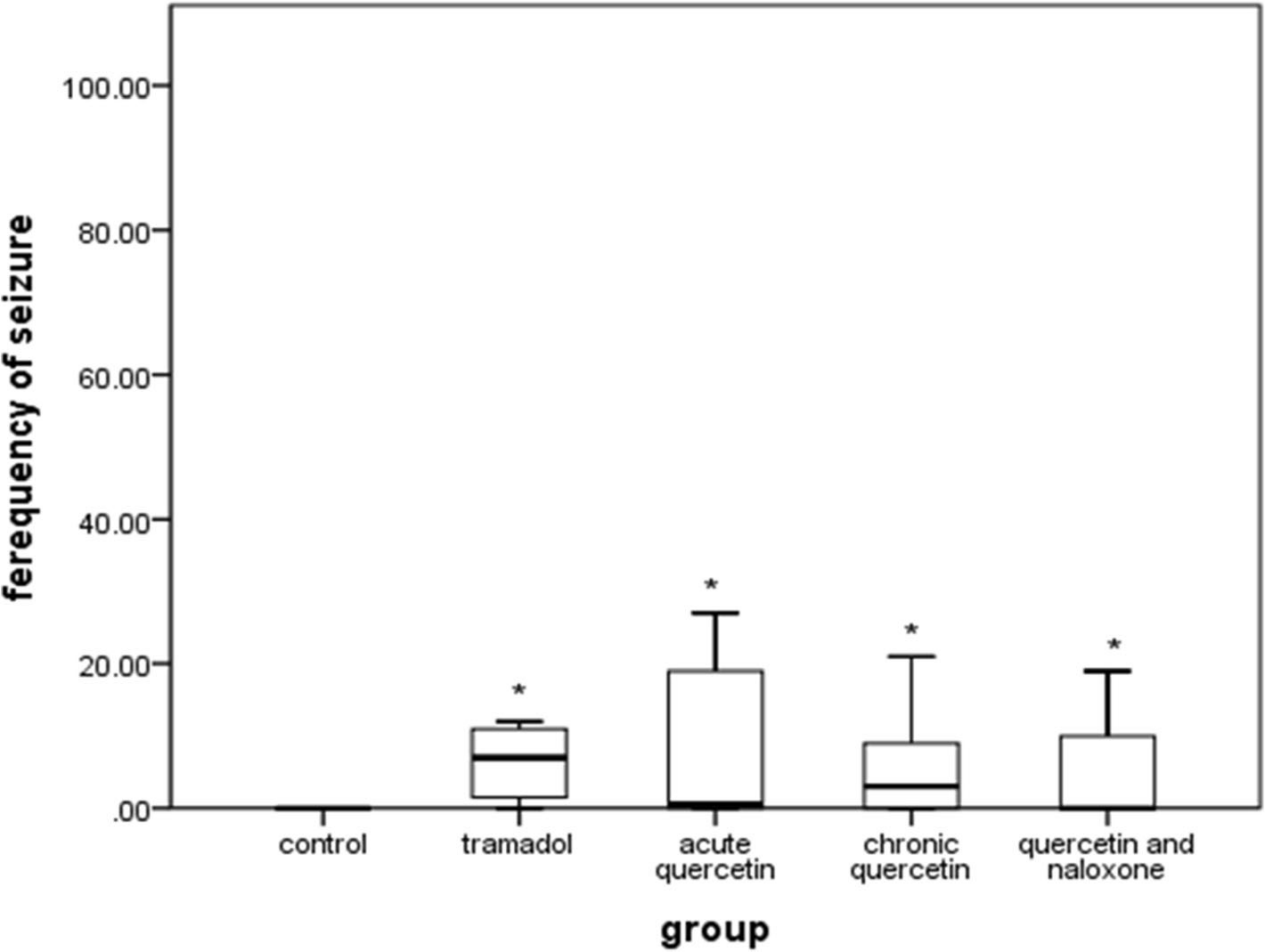
Comparison of the number of seizures in different groups. ^*^p < 0.05 compared to the Control group. Values are median and IQR

The results of this study showed that the number of seizures in all experimental groups was significantly higher than that of the control group (*p* < 0.05) (Fig. 1). However, none of the treatments significantly affected the number and severity of seizures compared to the tramadol group (p > 0.05).

### Hematologic and biochemical parameters

Chronic administration of quercetin resulted in an alteration of some blood parameters, such as reduced hemoglobin level and elevated platelet counts. In this regard, the chronic quercetin group’s median hemoglobin was significantly lower than that of the tramadol, single-dose quercetin, and quercetin plus naloxone groups. On the other hand, the mean platelet count was significantly higher in the chronic quercetin group than in others. Furthermore, the mean white blood count was higher in the single-dose quercetin group than in control. The administration of naloxone and a single dose of quercetin (100 mg) resulted in a significantly increased ALT level compared to the chronic quercetin group (Group IV). Further, the AST/ALT ratio was prominently higher in all groups than in control. However, the treatment did not affect the serum level of glucose and creatinine (Table 2).

**Table 2:** hematologic and biochemical parameters in different experimental groups

Variables	Control	Tramadol	Tramadol + single Quercetin	Tramadol + chronic Quercetin	Tramadol + single Quercetin + Naloxone
WBC10*3/μL	3.12 ± 0.9	4.02 ± 1.3	5.8 ± 1.4 *	5.2 ± 1.4	4.8 ± 1.3
RBC 10*6/μL	8.5 ± 0.9	8.98 ± 0.4	9.2 ± 0.3	8.34 ± 0.5	9.23 ± 0.4
HGB (g/dL)	14.9 [13.1, 15.25]	14.7 [14.25,15.75] †	15.3[14.85, 16.2] †	13.45 [13.05,14.05]	15.5 [14.97, 15.95] †
HCT (%)	45.5[40.85,46.9]	46.1 [43.65, 48.15]	47.1[45.1, 49.65]	41.15 [40.55,44.17]	47.15 [46.5, 48.7]
PLT (10*3/μL)	516.7 ± 21.7 †	478.2 ± 46.51 †	516.8 ± 69.5 †	727.16 ± 58.04	412.5 ± 38.31 †
MCV (μm*3)	51.8[51.2,52.7]	50.7 [50.6, 51.95]	51.4[50, 52.85]	50.05 [49.65, 51.9]	50.7 [50.47, 50.7]
MCHC (g/dL)	32.5[32,32.7]	32.7 [32, 32.95]	32.9[32.3, 33.05]	32.35 [31.82, 32.7]	32.7 [32, 33.17]
MCH (pg)	16.84 ± 0.2	16.62 ± 0.3	16.82 ± 0.4	16.28 ± 0.4	16.77 ± 0.4
LYM (%)	64.2 ± 7.7	66.2 ± 9.4	52.1 ± 14.6	52 ± 9.1	61.5 ± 9.7
MON (%)	0.62 ± 0.4	0.66 ± 0.4	1.44 ± 0.9	1.76 ± 0.5	1.47 ± 0.8
NEU (%)	32 ± 7.9	31 ± 10.5	44.2 ± 15.4	43.7 ± 8.1	34.5 ± 10.6
EOS (%)	2.4 ± 1.3	1.96 ± 2.3	0.9 ± 0.1	0.8 ± 0.6	1.57 ± 0.2
BAS (%)	1.7 [0.07, 2.1]	2.4 [0, 3.3]	3.2 [0.1, 5.5]	2.9 [0.4, 4.7]	2.9 [0.3, 3.5]
NEU/LYM ratio	0.51 ± 0.1	0.49 ± 0.2	0.94 ± 0.4	0.87 ± 0.2	0.59 ± 0.2
Glucose (mg/dl)	136.6 ± 18.08	154.3 ± 25.1	137.1 ± 18.3	123.3 ± 18.8	131.8 ± 12.4
Cr (mg/dl)	0.35 ± 0.04	0.36 ± 0.05	0.34 ± 0.08	0.32 ± 0.05	0.44 ± 0.21
AST (IU/L)	110.6 [91.6, 146.3]	177 [135.6, 203.5]	148.3 [138.2, 216.9]	157.05 [125.3, 182.6]	180.5 [145.6, 435.7]
ALT (IU/L)	59.4 ± 11	52.3 ± 11.4	39.1 ± 5.7	34.5 ± 16.3	79.2 ± 50.8†
AST/ALT ratio	1.9 [1.8, 2.1]	3.3 [2.6, 4.2] *	4.2 [3.1, 5.7] *	4.1 [3.1, 13.5] *	3.1 [2.7, 4.3] *

WBC, White Blood count; RBC, Red Blood count; HGB, hemoglobin; HCT, hematocrit; PLT, platelet; MCV, mean corpuscular volume; MCH, mean corpuscular hemoglobin; MCHC, mean corpuscular hemoglobin concentration; LYM, Lymphocytes; MON, Monocytes; NEU, Neutrophils; EOS, Eosinophils; BAS, Basophils; Cr, creatinine; AST, aspartate aminotransferase; ALT, alanine aminotransferase

Statistical comparison between groups was made using one-way ANOVA followed by Bonferroniposthoc or Kruskal Wallis test and Dunn-Bonferroni post hoc

* *p* < 0.05 significant as compared to the **control group**

† *p* < 0.05 significant as compared to **Tramadol + chronic Quercetin group**

### Inflammatory parameter and antioxidant enzyme activity of serum

The level of interleukin 6 (IL-6) did not significantly differ between groups. The serum concentration of TNF-a was significantly higher in chronic quercetin and quercetin-naloxone groups compared to the control group. The nitric oxide, TBARS, and SOD levels of the serum did not differ between the groups (*p* > 0.05) (Table 3).

**Table 3:** Inflammatory and oxidative stress parameters of serum in different experimental groups

Groups Variable	Control	Tramadol	Tramadol + single Quercetin	Tramadol + chronic quercetin	Tramadol + single Quercetin + Naloxone
NOx (μmol/l)	24.9 [19, 54.8]	51.4 [26.2, 73.9]	27.9 [11.16, 81.9]	22.1 [12.5, 27.6]	33.39 [12.18, 108.6]
SOD (U/ml)	439.5 ± 50.3	494.6 ± 99.1	527.9 ± 78.2	574.4 ± 76.5	441.5 ± 86.9
MDA (μmol/l)	2.15 ± 0.2	2.32 ± 0.3	2.39 ± 0.3	2.21 ± 0.4	2.5 ± 0.7
IL-6 (ng/L)	8.6 ± 3.4	11.4 ± 4.5	12.87 ± 4.4	13.01 ± 4	10.08 ± 1.7
Tnf-a (pg/mL)	10.32 ± 1.1	13.86 ± 4.3	13.7 ± 2.8	15.2 ± 1.3*	14.6 ± 0.8*

Statistical comparison between groups was made using one-way ANOVA followed by Bonferroniposthoc or Kruskal Wallis test and Dunn-Bonferroni post hoc

NOx: Nitric Oxide, SOD: Superoxide dismutase, MDA: Malondialdehyde, IL-6: interleukin-6, Tnf-a: Tumour Necrosis Factor-alpha

* *p* < 0.05 significant as compared to the **control group**

### The concentration of nitric oxide metabolites (NOx) in different tissues

Tramadol significantly increased NOx in the kidneys; however, it had no effect on the liver, heart, brain, or adrenal tissues. A quercetin injection (100 mg) along with tramadol for 14 days significantly decreased the liver’s nitric oxide level and restored the high NOx level following a tramadol injection in the kidney. There was a significantly higher NOx level in the acute quercetin and chronic quercetin groups than in the quercetin/naloxone group in the heart tissue. On the other hand, in the cortex, the nitric oxide level of the quercetin/naloxone group was significantly higher than in control, the acute quercetin, and chronic quercetin groups.

### Oxidative stress parameters of different tissues

The TBARS in adrenal tissue was significantly lower in a single dose and naloxone groups (III and V) than in control; it was also lower in the chronic quercetin group (IV) than in the control and tramadol groups. All of the three treatment methods in the adrenal tissue reduced the TBARS similarly. Further, the level of this factor was lower in the heart tissue of all groups than in the control, suggesting the antioxidant effects of all interventions. A significant rise of the TBARS in the liver tissue of the single-dose quercetin group (III) over tramadol and other treatment groups (II, IV, and V) indicates the pro-oxidant effects of acute quercetin. In kidney and brain tissue, the TBARS was similar among all groups. Tramadol administration with different treatments did not affect the SOD activity in different studied tissues (Table 4).

**Table 4:** Oxidative stress parameters of organs in different experimental groups

Groups Variable		Control	Tramadol	Tramadol + single Quercetin	Tramadol + chronic quercetin	Tramadol + single Quercetin + Naloxone
Liver	SOD (U/ml)	628.36 [604.3, 712.7]	673.3 [557.5, 686.7]	726.5[580.3, 898.5]	617.8 [594.6, 644.3]	759.4 [590.6, 855.48]
	NOx (μmol/l)	15.2 ± 1.8	14.5 ± 2.1	11.7 ± 2.4	10.6 ± 0.5*	12.9 ± 2.5
	MDA (μmol/l)	11.9 ± 3.9	8.7 ± 2.2	14.4 ± 2.1**,†,††	9.5 ± 1.5	8.9 ± 3.2
Kidney	SOD(U/ml)	832.2 ± 72.4	665.2 ± 78.7	758.7 ± 117.7	686.4 ± 83.7	741.9 ± 62.7
	NOx (μmol/l)	16.1 ± 2.9	21.3 ± 8.3*	16.8 ± 2.2	12.44 ± 1.6**	16.34 ± 1.2
	MDA (μmol/l)	20 ± 1.7	19 ± 2.6	21.5 ± 1.8	18.6 ± 2.1	20.7 ± 2.9
Heart	SOD(U/ml)	695.3 ± 183	792.3 ± 136.7	948 ± 95	725.3 ± 268	651 ± 120
	NOx (μmol/l)	11.2 [10.6,12.25]	11.06 [9.3, 13.1]	12.6 [10.5,15.6] ††	11.9 [11.9, 14.1] ††	9.5 [7.08, 10.7]
	MDA (μmol/l)	17.9 ± 4.2	10.2 ± 2.5*	9.4 ± 0.7*	8.6 ± 0.5*	8.9 ± 0.9*
Cerebellum	SOD(U/ml)	292.3 ± 21.26	319.8 ± 35.5	298.6 ± 19.7	278.9 ± 20.7	295.5 ± 27.06
	NOx (μmol/l)	8.08 ± 1.3	7.5 ± 0.6	7.64 ± 1.2	8.49 ± 1.4	8.01 ± 1.5
	MDA (μmol/l)	5.6 ± 1.3	4.5 ± 1.1	5.3 ± 0.4	5.8 ± 0.6	5.3 ± 1.5
Cortex	SOD(U/ml)	258.9 ± 15.32	275.7 ± 23.1	272.2 ± 17.5	260.1 ± 14.58	273.08 ± 10.11
	NOx (μmol/l)	3.46 ± 0.8	4.1 ± 2.2	2.65 ± 1.04††	1.53 ± 0.7††	6 ± 1.68*
	MDA (μmol/l)	4.86 [4.03, 5.6]	4.6 [4.2, 6.7]	4.9 [4.4, 6.1]	4.6 [4.2,6.1]	4.5 [4.4, 5.2]
Adrenal	SOD(U/ml)	577.3 ± 77.2	572 ± 50.5	576.6 ± 85.9	460.9 ± 48.4	584 ± 135
	NOx (μmol/l)	17.6 ± 3.5	17.1 ± 3.1	15.7 ± 4.2	15.3 ± 1.4	14.5 ± 2.8
	MDA (μmol/l)	4.5 [4.1, 4.8]	3.02 [2.6, 3.3]	2.4 [2.2,3.1] *	2.06 [1.9, 2.2] *,**	2.3 [2.1, 2.3] *

* *p* < 0.05 significant as compared to the **control group**

** *p* < 0.05 significant as compared to **tramadol group**

† *p* < 0.05 significant as compared to **Tramadol + chronic Quercetin group**

†† *p* < 0.05 significant as compared to **Tramadol + single Quercetin + Naloxone**

Statistical comparison between groups was made using one-way ANOVA followed by Bonferroni post hoc or Kruskal Wallis test and Dunn-Bonferroni post hoc

Values are mean ± SD or median [IQR]

## Discussion

### Biometrical data

In the current study, we investigated quercetin’s beneficial effects on tramadol overdose. Based on our information, this is the first study that assessed the effects of this flavonoid on tramadol intoxication. After the experimental period, the rats in the TRM + chronic quercetin group showed body weight loss and decreased heart weight. This result was reported in some previous studies (30). Differences in reports can be observed, however, depending on the experimental model and doses. One study investigated the effects of oral quercetin treatment on experimental renovascular hypertension in Wistar rats (10 mg/kg) within five weeks. According to the results, the treatment did not lead to kidney or heart weight variations compared to untreated controls (31). The examination did not reveal any alterations in food and water consumption, body weights, or organ weight of Swiss mice at any doses of flavonoids (30, 300, or 3000 mg/kg for 28 days) (32). One other study revealed that a 25 mg administration of quercetin/day within 28 days, as 0.5% of the diet, had an insignificant impact on Swiss mice’s body or organ weight gains compared to a control group (33). Organ weight can critically indicate an experimental compound effect, and a substantial difference in the weight of organs may happen in treated and control animals if no morphological variation occurs (34).

### Behavioral parameters

None of the treatments had significant effects on the number and severity of seizures. To our knowledge, quercetin effectiveness in tramadol-induced seizures has not been studied till now. However, its protective effects in other drug-induced seizures have been documented. For example, quercetin decreased kainic acid-induced seizure severity in a dose-dependent manner (12). The anticonvulsant effects of quercetin have been demonstrated at doses of 10 to 200 mg/kg in 6 Hz-induced convulsive seizures, the highest quercetin-related anticonvulsant activity associated with high plasma and cerebral concentrations (21). The exact anticonvulsant effects of quercetin and other flavonoids are not well-known, possibly mediated by their interactions with GABA receptors, glycine (35), acetylcholine (21), serotonin receptors (36), and adenosine (37).

### Hematologic and biochemical parameters

In our study, chronic administration of quercetin had effects on some hematological parameters, including decreased hemoglobin level and increased platelet count. It has been reported that the oral administration of quercetin decreased hemoglobin and hematocrit at 30, 300, or 3000 mg/kg within 28 days (32). Another study did not document any statistically significant changes in hematologic parameters along with chlorpyrifos in the quercetin treatment group compared to the control group (38). In contrast, some others reported beneficial effects of quercetin on RBC and hematocrit (39). The platelet rates’ values were significantly increased with simultaneous quercetin administration and polychlorinated biphenyls (40) or acute and sub-chronic manganese administration (41). Some studies have shown an inhibition of platelet aggregation following supplementation with flavonoids in vivo (42, 43). In an in vivo study, quercetin inhibited platelet activation by interacting with cell membranes by not scavenging free radicals (43). Other animal studies have shown unaltered blood platelet counts in treatment with quercetin (44, 45). Also, tramadol is reported to cause experimental animals to experience hepatotoxicological variation in acute and chronic administration (46, 47). The present study results indicated that TRM led to the increased AST/ALT ratio in the serum related to male rats. A muscle injury due to tramadol-induced seizures should be considered for an increase in these enzymes.

### Inflammatory and oxidative stress parameters

The levels of some oxidative stress markers in the tramadol group were no different than in the control group. Some studies have shown that chronic and acute administration of tramadol can lead to increased oxidative stress in the body (7, 10, 48–50). Some others have reported lower MDA levels in the tramadol receiving groups than in the control group. This confirms the hypothesis, which claims that tramadol reduces the effects of oxidative stress via peroxyl radicals scavenging. Also, in that study, the SOD data supported the possibility of a tramadol antioxidant effect when the drug is used during the pre-ischemic period (51). The differences in study design and experimental models may explain the discrepancies. In this study, the animals received acute on chronic administration of tramadol. The initial dose of tramadol may cause tolerance (52) and reduce adverse effects. Therefore, these results indicate the need for further studies.

The MDA content can be used to estimate the extent of lipid peroxidation (53). In the current study, all types of quercetin administration effectively reduced the MDA content of adrenal tissue. The increase of malondialdehyde in the liver in the acute quercetin group, which has not been seen in the chronic quercetin and naloxone-quercetin groups, presumably results from liver enzyme induction quercetin or positive effects of naloxone. However, we have not undertaken biochemical studies to verify this assumption. Our results also showed that chronic administration of quercetin in tramadol overdose decreased NOx formation in the liver, cortex, and kidneys, but not in the heart. As a member of the flavonoids family, Quercetin is demonstrated to function as a scavenger of both different reactive oxygen species and antioxidant activity (54). These support previous studies on different induced oxidative stressors (55). Quercetin administered orally and intraperitoneally can decrease the MDA level, which is induced by numerous toxins in rats (54). This ameliorative impact might be because of their greater diffusion into membranes, enabling them to scavenge free radicals at various sites (56) and regenerate exogenous and endogenous antioxidants, such as vitamins C and E as well as glutathione (45).

Cellular studies have revealed that quercetin can generate antioxidant and pro-oxidant impacts under certain conditions (57). It has been argued that the cellular oxidative balance and glutathione (GSH) content have an essential role in determining these effects (57). The quercetin pro-oxidant activity might have an auto-oxidizing capacity or might be able to convert to ortho-semiquinone and ortho-quinone/quinone methide intermediates through enzymatic oxidation, which may contribute to ROS generation and GSH depletion (58, 59). The activities of antioxidant enzymes have also been reported to be inhibited or increased by quercetin (44–46), and it depends on the experimental model (60–62) or the dose of the drug. Nevertheless, the significance of this mechanism has not been thoroughly resolved in vivo. It has been demonstrated that quercetin concentrations of up to 10 μM can protect fibroblasts from oxidative stress-induced damage and that following pre-treatment with quercetin concentrations of 30 μM, cytotoxicity occurred instead (63).

Additionally, the results revealed that the naloxone and quercetin co-treatment has toxic effects in cortex and liver tissues, compared to chronic quercetin administration, suggesting that naloxone has an adverse effect on some oxidative and liver function parameters. Most guidelines recommend naloxone as the first method of management in opioid poisoning, and there are some controversies between studies regarding using naloxone in tramadol overdose because of the possible risk for seizure (64–68).

In contrast to our results, in some reports (69), the quercetin anti-inflammatory effect was correlated with its free radical scavenging and anti-oxidative features; thus, eliminating reactive oxygen species can simultaneously prevent oxidation and inhibit inflammation. It has been noted that reactive oxygen species and the oxidation process contribute to the inflammatory responses via activating transfer variables, including nuclear factor-κ-gene binding (NF-κB) (70). Further, NF-κB has the capacity to induce TNF-α cytokine production (71). An in vivo study on mice reported a reduced inflammatory gene expression using the quercetin enriched diet (72). However, studies performed on healthy human volunteers with the help of quercetin demonstrated no effects on inflammatory agents existing in human blood (73).

### Limitation

This study has some limitations that should be taken into consideration for interpreting the data. First, based on the animal study design, the findings can only be indicative rather than representative of humans’ complicated clinical situations. However, our model showed tramadol-induced seizures similar to that documented in humans (74–76). Second, the dose dependence effects of quercetin and its histopathological alteration were not investigated in this study. Third, we did not assess possible mechanisms by which quercetin alters hematologic, oxidative, and inflammatory parameters. Fourth, the seizures parameters were evaluated using visual assessment; other assessment methods are suggested for future studies. Moreover, further experimental research is required to reveal the effectiveness of quercetin in a different type of tramadol administration and the molecular pathways of quercetin’s effects on tramadol overdose with more powerful methods that provides more robust results.

## Conclusion

None of the treatments had significant effects on the number and severity of seizures. Within the limitation of the animal model study, it can be concluded that acute on chronic administration of tramadol induced a significant increase in AST levels. Besides, chronic supplementation with quercetin during the experimental period decreased oxidative/nitrosative stress in the heart, adrenal, liver and kidney tissues. On the other hand, it imposed thrombocyte imbalance. Quercetin and naloxone decreased oxidative stress in the heart and adrenal tissue; however, they adversely affected cortex tissue. Contrary to the inconsistent effect of quercetin on liver tissue, a single dose of quercetin similar to its chronic administration reduced cardiac oxidative stress.

## Data Availability

The datasets are available from the corresponding author on formal and logical requests.

## Abbreviations

TRM: Tramadol
ROS: reactive oxygen species
NF-κB: nuclear factor-kappaB
IP: intraperitoneal
TBA: thiobarbituric acid
SOD: superoxide dismutase
ANOVA: one-way analysis of variance
GSH: glutathione
NF-κB: Nuclear factor-kappa B
CBC: complete blood count
WBC: White Blood count
RBC: Red Blood count
HGB: hemoglobin
HCT: hematocrit
PLT: platelet
MCV: mean corpuscular volume
MCH: mean corpuscular hemoglobin
MCHC: mean corpuscular hemoglobin concentration
LYM: Lymphocytes
MON: Monocytes
NEU: Neutrophils
EOS: Eosinophils
BAS: Basophils
Cr: creatinine
AST: aspartate aminotransferase
ALT: alanine aminotransferase
NOx: Nitric Oxide metabolites (nitrate+ nitrite)
SOD: Superoxide dismutase
MDA: Malondialdehyde
IL-6: interleukin-6
Tnf-a: Tumour Necrosis Factor-alpha
